# Acute exacerbation in chronic bird fancier's lung with pleuroparenchymal fibroelastosis

**DOI:** 10.1002/rcr2.693

**Published:** 2020-11-20

**Authors:** Keishi Sugino, Hirotaka Ono, Natsumi Watanabe, Seiji Igarashi, Akira Hebisawa, Eiyasu Tsuboi

**Affiliations:** ^1^ Department of Respiratory Medicine Tsuboi Hospital Koriyama City Japan; ^2^ Department of Pathology Tsuboi Hospital Koriyama City Japan; ^3^ Department of Histopathology Asahi General Hospital Chiba Japan

**Keywords:** Bird dropping, bird fancier's lung, feather, hypersensitivity pneumonitis, pleuroparenchymal fibroelastosis

## Abstract

A 71‐year‐old non‐smoker woman was admitted to our hospital complaining of a six‐month history of dry cough. She had kept java sparrow for nine years and has been raising budgerigars for the previous eight months. High‐resolution computed tomography (HRCT) images of the chest revealed reticulonodular lesions predominantly in the bilateral upper lobes. Surgical lung biopsy specimens showed non‐caseous epithelioid cell granulomas in the alveolar spaces, including irregular and centrilobular fibrosis with pleuroparenchymal fibroelastosis. When she started using a duck feather duvet at home, she developed dyspnoea and chest HRCT abnormalities progressively deteriorated. The results of precipitation of antibodies against duck feather, java sparrow, and budgerigars dropping extracts were positive in sera. Consequently, the patient was diagnosed as having chronic bird fancier's lung with acute exacerbation caused by the use of a feather duvet. After combination treatments with corticosteroid and cyclosporine, her respiratory symptoms and reticulonodular shadow immediately improved.

## Graphical abstract

This is a case of chronic bird fancier's lung (BFL) (java sparrow and budgerigars) presenting with acute exacerbation due to exposure to feather duvet. Chronic BFL occasionally can associate with pleuroparenchymal fibroelastosis (PPFE). Searching for precipitins against antigens of bird droppings and feather is useful for our clinicians to make a diagnosis of BFL.

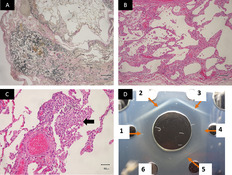

## Introduction

Bird fancier's lung (BFL) is observed in individuals who develop a hypersensitivity response to avian droppings and antigens in feathers [1]. Non‐idiopathic pleuroparenchymal fibroelastosis (PPFE) has been increasingly reported in association with several interstitial lung diseases, including idiopathic pulmonary fibrosis (IPF), hypersensitivity pneumonitis (HP), and familial forms of pulmonary fibrosis [2].We report a patient with chronic BFL (java sparrow and budgerigars) with PPFE presenting with acute exacerbation caused by the use of a feather duvet.

## Case Report

**Figure 1:**
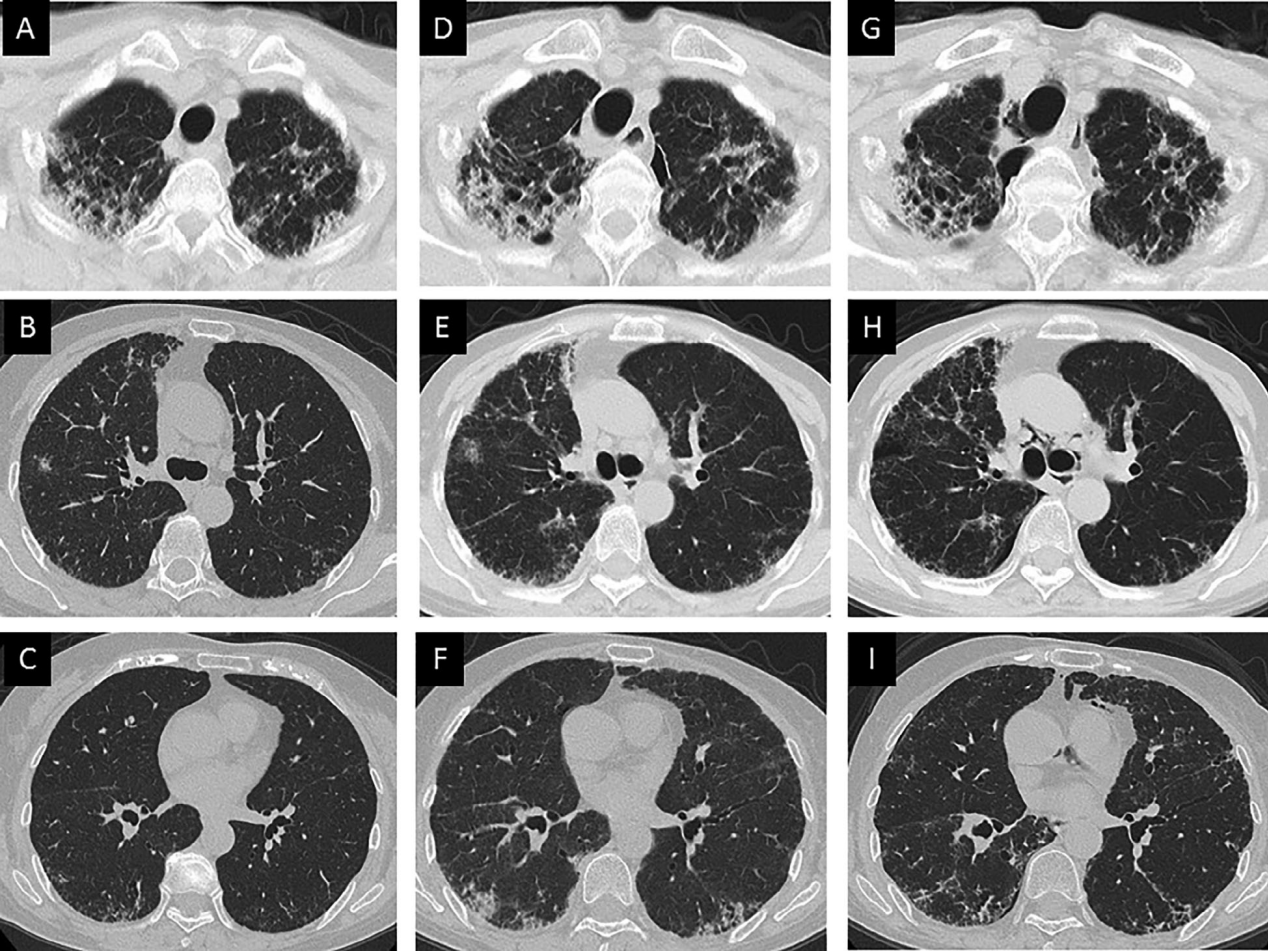
(A–C) Chest high‐resolution computed tomography (HRCT) images revealed patchy bilateral ground‐glass opacities (GGO), nodular areas of consolidation, and multiple small nodules in the bilateral upper lobes predominance, in addition to bilateral dense subpleural consolidation and irregular septal thickening at the level of the lung apices. (D–F) Twelve months after the initial visit, reticulonodular lesions in both lung and coarse fibrosis predominating in the peripheral upper lobes worsened. (G–I) Three months after treatment with corticosteroid and cyclosporine, reticulonodular lesions in both lung and bilateral subpleural reticulation at the level of the lung apices were partially improved. Pneumomediastinum and minor pneumothorax were seen.

**Figure 2:**
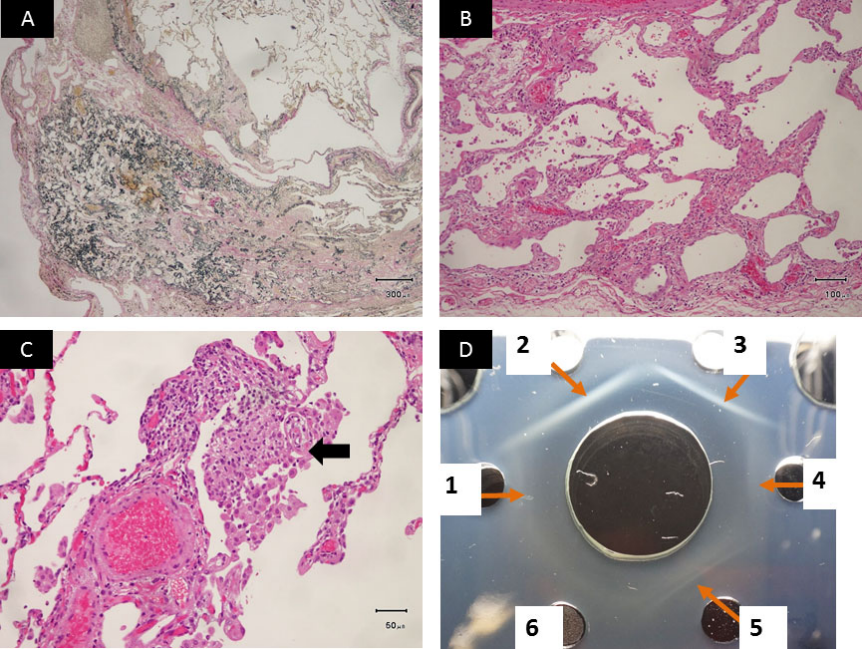
Histopathological findings of lung biopsy specimens obtained by video‐assisted thoracic surgery (VATS). (A) Low magnified microscopic appearance of the right upper lobe revealed thickened fibrous pleura and subpleural fibroelastosis (elastic van Gieson stain) (1 scale = 300 μm). (B) Low magnified microscopic appearance of the right lower lobe showed diffuse and temporally uniform interstitial fibrosis with chronic inflammatory cells infiltration (haematoxylin–eosin stain) (scale bar = 100 μm). (C) High magnified microscopic appearance of the right lower lobe. Note poorly defined non‐caseous epithelioid cell granulomas in the alveolar spaces (arrow) (haematoxylin–eosin stain) (scale bar = 50 μm). (D) The results of precipitation of antibodies against duck feathers (2, 3), java sparrow (4), and budgerigars dropping extracts (5) were positive in sera. 1: Precipitation line between pigeon dropping extracts and patient sera. 2: Precipitation line between duck feather made in Japan and patient sera. 3: Precipitation line between duck feather made in Taiwan and patient sera. 4: Precipitation line between java sparrow dropping extracts and patient sera. 5: Precipitation line between budgerigars dropping extracts and patient sera. 6: Negative control between grass hay and patient sera.

A 71‐year‐old non‐smoker woman was admitted to our hospital for dry cough with onset at six months before admission. She had kept java sparrow for nine years and has raised budgerigars for the past eight months. Laboratory evaluation revealed elevated serum levels of surfactant protein‐D (SP‐D: 333 ng/mL). Arterial blood gas analysis showed a pH of 7.40, partial pressure of carbon dioxide (PaCO_2_) of 42.5 Torr, and partial pressure of oxygen (PaO_2_) of 77.9 Torr on room air. The pulmonary function test revealed normal range (forced vital capacity (FVC) of 2.31 L and 98.7% of predicted, forced expiratory volume in 1 sec (FEV_1_) of 2.17 L and 117.9% of predicted) with diffusion capacity for carbon monoxide (DL_CO_ of 12.61 mL/min/mmHg, 87.9% of predicted). High‐resolution computed tomography (HRCT) images of the chest revealed patchy bilateral ground‐glass opacities (GGO), nodular areas of consolidation, and multiple small nodules in the bilateral upper lobes predominance, in addition to bilateral dense subpleural consolidation and irregular septal thickening at the level of the lung apices (Fig. 1A–C). Examination of bronchoalveolar lavage fluid (BALF) revealed alveolar macrophages, 68%; lymphocytes, 29.2%; neutrophils, 2.8%; and eosinophils, 0%, with increased total cells and a normal CD4/CD8 ratio of 1.6. Cultures of sputum and BALF were negative for fungal, bacterial, or mycobacterial pathogens. The lung biopsy specimens of the right upper and lower lung segments, obtained by video‐assisted thoracic surgery (VATS), revealed poorly defined non‐caseous epithelioid cell granulomas in the alveolar spaces with alveolitis, as well as irregular and centrilobular fibrosis with PPFE (Fig. 2A–C). After undergoing VATS, she started using a duck feather duvet at home. As a result, she had developed progressive dyspnoea on exertion and dry cough. Moreover, chest HRCT images progressively deteriorated (Fig. 1D–F). Laboratory findings revealed elevated Kreb von den Lungen‐6 (KL‐6) (1130 U/mL) and SP‐D (713 ng/mL). The pulmonary function test revealed restrictive impairment (FVC of 1.40 L, 60.3% of predicted) with decreased diffusion capacity (DL_CO_ of 7.94 mL/min/mmHg, 59.6% of predicted). The results of precipitation of antibodies against duck feather, java sparrow, and budgerigars dropping extracts were positive in sera (Fig. 2D). Consequently, the patient was diagnosed as having chronic BFL with PPFE due to java sparrow and budgerigars presenting with acute exacerbation caused by the use of a feather duvet. After avoidance of breeding of budgerigars and use of a duck feather duvet, her general conditions led to mild improvement. We initiated a combination therapy of corticosteroid (20 mg/day) and cyclosporine (150 mg/day). After three months of receiving this combination therapy, her clinical condition gradually improved. The values of 6‐minute walking distance (6MWD), lowest peripheral capillary oxygen saturation (SpO_2_), %FVC, %DL_CO_, and PaO_2_ increased and those of KL‐6 and SP‐D decreased. In addition, reticulonodular lesions on chest HRCT scans were associated with a trend towards improvement (Fig. 1G–I). No serious side effects were reported in the patient.

## Discussion

Patients with chronic insidious BFL tend to be diagnosed as having idiopathic interstitial pneumonias, including IPF. Craig et al. reported that high levels of bird antigen can be detected for prolonged periods of time after bird removal and environmental clean‐up [1]. In fact, Inase et al. reported a case of chronic BFL (breeding budgerigars and mynahs) presenting with acute exacerbation due to exposure to feather duvet [3]. These findings support the fact that BFL is caused by not only direct exposure (breeding birds), but also unrecognized exposure (feather products, wild birds, and next‐door neighbour's birds) regardless of these antigens' avoidance. Several researchers have also reported some patients with PPFE had concurrent histological patterns suggestive of HP [2, 4]. As reported by Jacob et al., marked PPFE was identified in 23% of patients with HP [5]. Therefore, PPFE may represent a progressive fibrosing immune‐mediated response due to recurrent infections, inhaled antigen, or allergen. We assumed that the histological findings indicated the overlapping of the acute or subacute phase such as alveolitis and the chronic phase such as PPFE with centrilobular fibrosis. In conclusion, we believe that our patient had chronic BFL with PPFE due to java sparrow and budgerigars presenting with acute exacerbation caused by the use of a feather duvet.

### Disclosure Statement

Appropriate written informed consent was obtained for publication of this case report and accompanying images.
